# Effect of Beta Particles Spectrum on Absorbed Fraction in Internal Radiotherapy

**DOI:** 10.22038/AOJNMB.2018.11610

**Published:** 2019

**Authors:** Marjan Hashempour, Mahdi Ghorbani, Ernesto Amato, Courtney Knaup

**Affiliations:** 1 Physics Group, Faculty of Basic Sciences, Hakim Sabzevari University, Sabzevar, Iran; 2 Biomedical Engineering and Medical Physics Department, Faculty of Medicine, Shahid Beheshti University of Medical Sciences, Tehran, Iran; 3 Department of Biomedical and Dental Sciences and of Morphofunctional Imaging, University of Messina, Messina, Italy; 4 Comprehensive Cancer Centers of Nevada, Las Vegas, Nevada, USA

**Keywords:** bsorbed fraction, nalytical function, Beta emitters, nternal radiotherapy, Monte Carlo simulation

## Abstract

**Objective(s):**

The purpose of this research is to study the effect of beta spectrum on absorbed fraction (*ϕ*) and to find suitable analytical functions for beta spectrum absorbed fractions in spherical and ellipsoidal volumes with a uniform distribution for several radionuclides that are commonly used in nuclear medicine.

**Methods:**

In order to obtain the beta particle absorbed fraction, Monte Carlo simulations were performed by using the MCNPX code. The validation of the simulations was performed by calculating the absorbed fractions in spheres and comparing the results with the data published by other investigators. The absorbed fractions were calculated and compared by using an actual beta energy spectrum with those obtained through the mean beta energy of ^14^C, ^199^Au, ^177^Lu, ^131^I, ^90^Sr, ^153^Sm, ^186^Re, ^32^P, ^90^Y, ^38^Cl and ^88^Rb radionuclides.

**Results:**

The maximum difference between the absorbed fractions for beta particles accounting for the whole beta spectrum of all the considered nuclides was 29.62% with respect to the mean beta energy case. Suitable analytical relationships were found between the absorbed fraction and the generalized radius, and the dependence of the fitting parameters from beta spectrum energy was discussed and fitted by appropriate parametric functions.

**Conclusion:**

The results allowed the calculation of the absorbed fractions from the above stated beta sources uniformly distributed in spherical and ellipsoidal volumes of any ellipticity and volume, in a wide range of practical volumes that are not only used for internal dosimetry in nuclear medicine applications, but also in radiological protection estimates of doses from internal contamination.

## Introduction

Beta-emitting radionuclides are extensively used for the treatment of tumors and various diseases in the form of internal radiotherapy as well as for tumor imaging (1). Radiopharmaceuticals labeled with ^177^Lu, ^131^I, ^90^Sr, ^153^Sm, ^186^Re, ^32^P, and ^90^Y are used for the treatment of various types of cancers such as the palliation of bone metastases and ocular surface tumors, liver tumors, breast cancer, and for metastatic prostate cancer (2-8). The knowledge of radiation absorbed fraction, i.e. the fraction of emitted energy which is absorbed within the target volume, is an essential issue for both radionuclide internal dosimetry and radiological protection (9).

In the approach presented by the Medical Internal Radiation Dose (MIRD) Committee, beta absorbed fraction is considered equivalent to unity in large organs, but in smaller regions, it is less than unity and differs depending on the size and the geometry of the target organ (10). The absorbed fraction tends to be unity when the target dimensions are greater than the range of the beta emitted by the source. On the other hand, when this condition is not satisfied the absorbed fraction may be lower and its dependence on the target dimensions and beta energy cannot be overlooked (11). 

In nuclear medicine, the absorbed fraction is an important parameter for calculating the absorbed dose. One of the factors that affect the absorbed fraction is the energy emitted by the source. In dosimetric calculations with commercially available software (12), only the monoenergetic spectrum may be taken into account. When the monoenergetic beta spectrum is used, the quantity of the energy absorbed by the target differs from the actual value (11). This causes discrepancies in dose calculation. Since the adopted criteria in nuclear medicine planning are based on tolerance doses, it highlights the importance of critical organ doses that must be estimated accurately.

A number of the pamphlets published by the MIRD committee have made wide use of the Monte Carlo simulation to calculate the absorbed fractions for beta emitting sources uniformly distributed in organs. Akabani *et al.* (11) estimated the beta absorbed fractions in small spherical volumes of tissue for a number of radionuclides by using mean energy and the entire beta spectrum. They showed that for volumes with radii greater than the range of the electrons, the use of mean beta energy of the beta spectrum gave a good approximation to the absorbed fraction, when compared to the results acquired by the extended beta spectra of the radionuclides. Additionally, they found the absorbed fractions in the source region for various radionuclides as a function of the surface-to-volume ratio.

A spherical model is employed to model nodules and other small regions in human dosimetry, as well as for whole non-human organisms in an environmental radioactivity pollution assessment. Additionally, generalization to ellipsoids is favorable, when the assumption of a spherical shape for an ellipsoidal structure leads to in accurate dosimetric evaluations of the order of several percent, depending on the dimensions, the degree of non-sphericity, and the radionuclide emission spectrum. Amato *et al.* (13) used the Geant4Monte Carlo code to calculate the absorbed fractions for uniformly distributed^ 199^Au, ^177^Lu, ^131^I, ^153^Sm, ^186^Re and ^90^Y beta emitters, using proper extended beta energy spectra in ellipsoidal volumes of soft tissue. They proved that the absorbed fraction was a function of the generalized radius and calculated the *ρ*_0_ and *s *parameters of the analytical fit for each radionuclide. Based on the results, *ρ*_0_ (the cut-off radius) was approximately proportional to the average beta energy of the radionuclide, but there was no relationship between the exponential term *s* and the average beta energy. They presented a mean value of 1.13 for the *s* parameter for radionuclides that were different from those included in their study. Subsequently, Amato *et al*. (9) calculated the absorbed fractions inhomogeneous ellipsoid volumes made from soft tissue by considering monoenergetic electrons of 10 energies: 10, 20, 50, 70, 100, 200, 500, 700, 1000 and 2000 keV. They introduced analytical functions for the relationship between *s* and *ρ*_0_, and the mean electron energy. They showed that dose calculation in the case of extended beta spectra can be evaluated through integration. In another study, Mowlavi *et al.* (10), based on Monte Carlo simulations with MCNP code, found analytical functions for the beta and gamma absorbed fractions of ^131^I in spherical and ellipsoidal volumes of soft tissue.

The MIRD method for dose calculation can be used when the source organ and the target organ are different from each other or when they are the same (10). The present study investigates the effect of discrete spectrum of beta emitters on the absorbed fraction. Furthermore, suitable analytical functions are found for the absorbed fractions obtained by the application of beta discrete spectrum in spherical and ellipsoidal volumes with the uniform distribution of ^14^C, ^199^Au, ^177^Lu, ^131^I, ^90^Sr, ^153^Sm, ^186^Re, ^32^P, ^90^Y, ^38^Cl and ^88^Rb radionuclides, when these volumes are defined as sources.

## Methods

**Figure 1:**
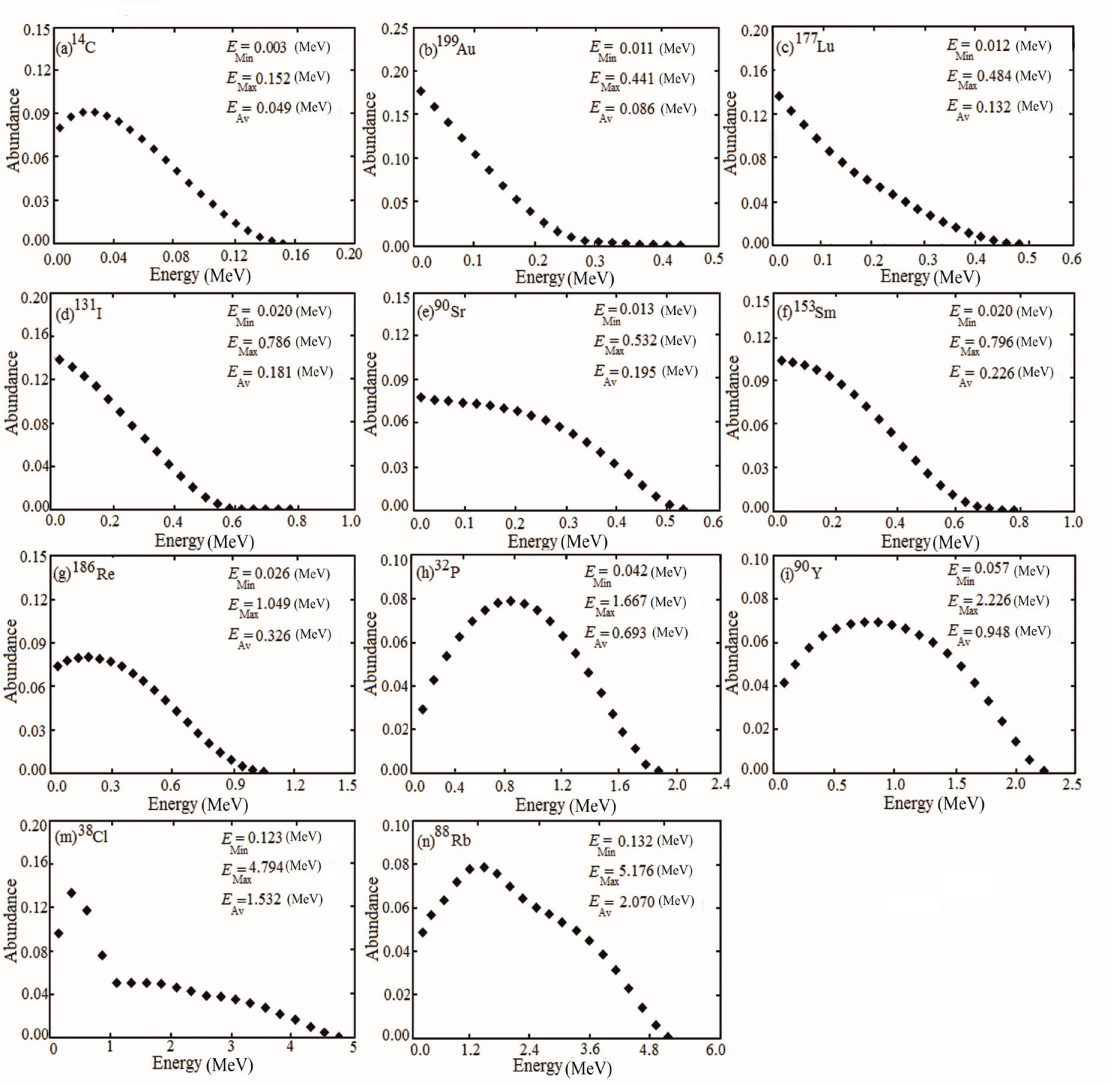
Energy spectrum, minimum, maximum and mean energy of beta particles for^ 14^C, ^199^Au, ^177^Lu, ^131^I, ^90^Sr, ^153^Sm,^ 186^Re, ^32^P, ^90^Y, ^38^Cl and^ 88^Rb radionuclides (14)

The influence of the discrete spectral definition of beta energy on the absorbed fraction was evaluated when the source organ coincided with the target organ. Absorbed fractions (self-absorption) were calculated for^14^C, ^199^Au, ^177^Lu, ^131^I, ^90^S, ^153^Sm, ^186^Re, ^32^P, ^90^Y, ^38^Cl and ^88^Rb beta emitter radionuclides, uniformly distributed in spherical and ellipsoidal volumes of soft tissue. The beta energy discrete spectra of these radionuclides are illustrated in Figure 1 (14). Soft tissue with a density of 1.04 g/cm^3^ was adopted as the constituent material of all the target volumes (15).

All the simulations were performed by the MCNPX (version 2.7.0) Monte Carlo code. In the first part of this investigation, the validation of the simulations was performed by calculating the absorbed fraction of ^90^Y radionuclide with volumes ranging from 0.004 cm^3^ to 100 cm^3^ in spherical volumes and by comparing the results with the data published by other investigators (11, 13). During the validation step, the volumes of the spheres were rounded up to one decimal point. These validations were performed only for ^90^Y because there was no detailed data on the absorbed fraction for other beta emitter radionuclides. In the second part of this investigation, the absorbed fractions obtained by applying the average and the discrete spectral beta energies in spherical and ellipsoidal volumes for ^14^C, ^199^Au,^177^Lu, ^131^I, ^90^Sr,^153^Sm,^186^Re,^32^P,^90^Y,^38^Cl and^ 88^Rb radionuclides were calculated and the results were compared with each other. Finally, suitable analytical functions for the beta discrete spectrum absorbed fractions were presented.

***Validation of Monte Carlo simulations***

The validation of the Monte Carlo simulations was performed by calculating the absorbed fraction obtained by the average and the discrete spectrum beta particles of ^90^Y radionuclide in spherical volumes and comparing the results with the data previously published by Akabani*et al*. (11) and Amato *et al*. (13). For this purpose, spheres with volumes ranging from 0.004 cm^3^ to 100 cm^3 ^were simulated and the *F8 tally was scored. The *F8 tally considers the energy deposition of particles on their transport path when photons and electrons interact with matter, rather than the instantaneous energy deposition at the position of the interaction. The simulations were performed for up to 10^6^ particle histories. In these calculations, the energy cut off was 1 keV for beta particles.

***Effect of discrete spectral definition of beta energy on absorbed fraction***

According to the MIRD approach, the mean absorbed dose from a monoenergetic emission of a radiation *j* to the target volume is calculated as:

$\bar{D_{j}}=\frac{\overset{\text{\textasciitilde{}}}{A}\mathrm{n E\emptyset}}{m}$

 (1)

Where $\overset{\text{\textasciitilde{}}}{A}$ is the time-integrated activity (Bqs); *m* is the target mass (in terms of g or kg), *E* is the particle energy (MeV), *n* is the emission probability per disintegration, and ϕ is the absorbed fraction (16).

The absorbed fraction (ϕ) depends on the emission type, energy, and also the geometry and the specification of the source and the target tissue. These factors used for dose calculation when either the source organ is different from the target organ (i.e. cross-organ dose) or when they are the same (i.e. self-dose) (17). The dose for an extended beta spectrum is obtained by the following integration:

D=A~m∫0EMdn(E)dE E∅(E)dE

 (2)

**Figure 2:**
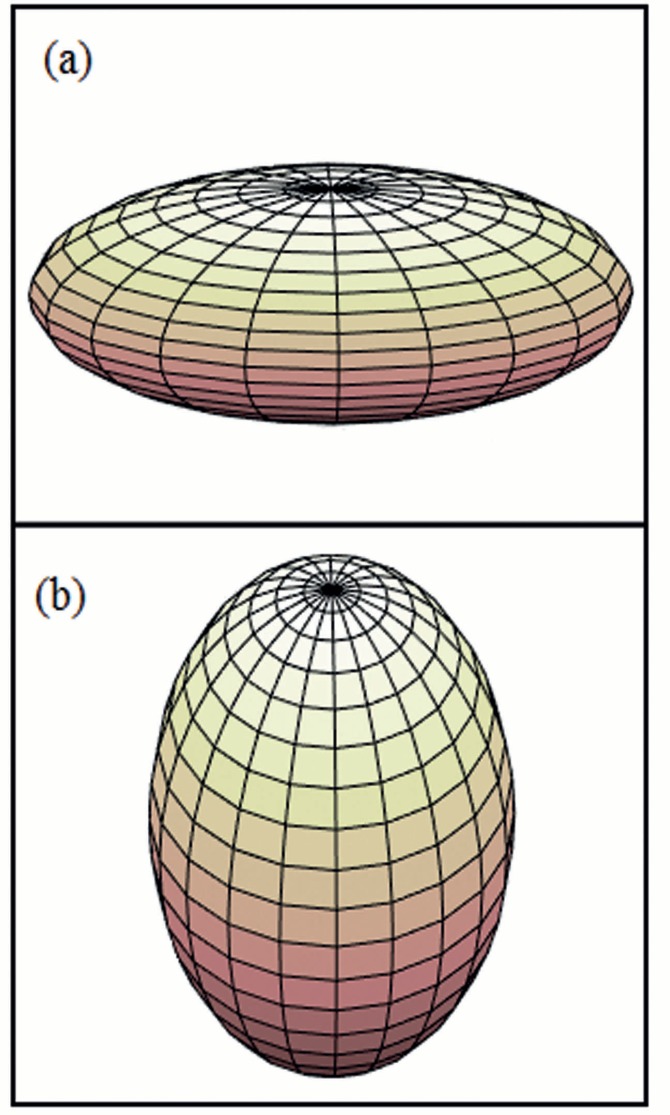
A schematic figure of prolate ellipsoid with (a) *a*=*b*=c 2 and oblate ellipsoid with (b)* a*=*b*=2*c*

Where* E* is the particle energy, d*n*/d*E* is the differential emission probability of the radionuclide and *E*_M_ is the most probable energy emitted. For the determination of the absorbed fraction by the entire beta spectrum by, using the *F8 tally, nine volumes ranging from 0.01 cm^3^ to 250 cm^3^ were simulated. Each volume had a different geometry: spherical, prolate ellipsoid with (*a*=*b*=c 2) and oblate ellipsoid with (*a*=*b*=2*c*),where *a*, *b* and *c* are the coordinate axes. The prolate ellipsoid with (*a*=*b*=c 2) and the ellipsoid with (*a*=*b*=2*c*) are illustrated in Figure 2. It should be noted that in this study, discrete spectrum was used for beta particles. 

 The physical characteristics, including elemental compositions and densities, of the soft tissue used in these simulations are listed in Table 1. The absorbed fraction was assessed by using the following equation:

$\emptyset_{\beta }=\frac{E_{\beta }}{E_{T}}$

 (3)

*E*_β_ is the energy of the beta particle absorbed in the target and *E*_T_ is the energy of the beta particle. To compare the absorbed fractions of the various radionuclides as a function of the target dimension, for a generic ellipsoidal shape, the “generalized radius” is defined as (13):

$\rho =3\frac{V}{S}$

 (4)

Where *V* is the target volume and *S* is its surface. For ellipsoidal shapes, *V* and *S *are obtained from:

$V=\frac{4}{3}\mathrm{\pi abc and S}\approx 4\pi {\left(\frac{\left({\mathrm{ab}}^{P}\right)+\left({\mathrm{ac}}^{P}\right)+({\mathrm{bc}}^{P})}{3}\right)}^{\frac{1}{p}}$

(5)

Where *p* ≈ 1.6075, and *a*, *b* and *c* are the semi-axes. For a sphere, the generalized radius is equal to the radius of the sphere.

In the present study, a new relationship was proposed between ϕ and *ρ*, by fitting the ϕ (*ρ*) graph versus *ρ*:

$\emptyset ={\left(1+\frac{\rho_{0}}{\rho^{s}}\right)}^{-1}-p$

 (6)

The *s, ρ*_0_ and *p* parameters are the fitting parameters. The fittings were performed by using MATLAB (The Math Works, Inc. Corporation, Version R 2015 b) 

In 2000, FDA (the U.S. Food and Drug Administration) rewrote the MIRDOSE code in order to increase the accuracy in dosimetric calculations. This new code was renamed OLINDA/EXM (18). It is employed in nuclear medicine departments for standardization and automation of internal dose calculations. The OLINDA/EXM code includes a sphere model that assumes isolated unit density (19).

In the present study, the OLINDA/EXM sphere model was run for three nuclides of ^32^P, ^90^Y, and ^38^Cl and a non-linear interpolation was made between the values in order to determine the OLINDA/EXM estimations at the exact volumes. Then, the analytical method provided in this paper was implemented on an electronic spreadsheet for the three nuclides, and self-doses were determined for the same spheres. The calculation were based on the spectra taken from the database by RADAR working group (20), which has the same reference as that of the OLINDA/EXM software.

## Results

Table 2 presents, the comparison of the absorbed fractions related to the mean and discrete spectral beta energies for ^90^Y radionuclide in the spheres obtained in this study with the results published in previous studies (11, 13). The deviation between the absorbed fraction values, *σ *(%), is defined as:

$\sigma =100\times (\emptyset -\emptyset_{\mathrm{Ref}})$

(7)

**Figure 3:**
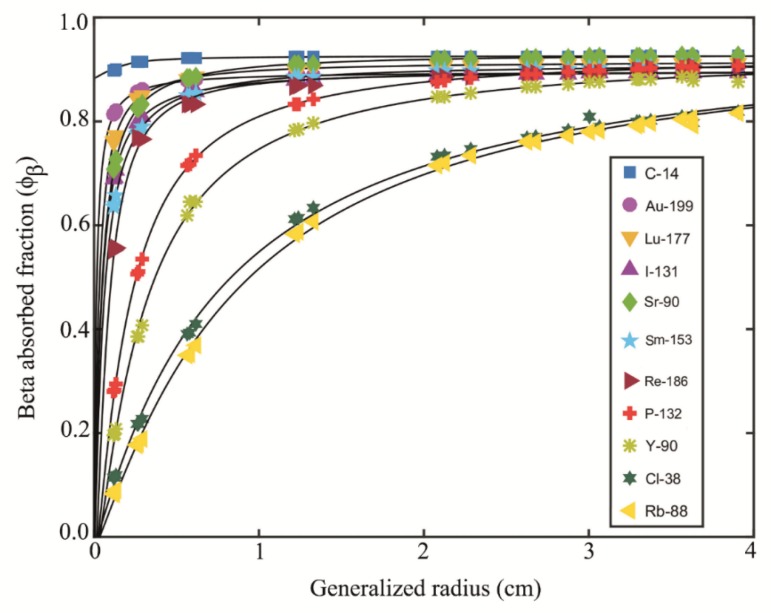
Absorbed fractions as a function of generalized radius obtained by spectral beta energies for various radionuclides

The differences in the absorbed fractions for ^90^Y radionuclide between the present study and previous studies ranged from 1.00% to 5.00%. For the purpose of comparison, the absorbed fractions obtained by the average energy and the discrete spectrum energies in spherical and ellipsoidal volumes with a uniform distribution of ^14^C, ^199^Au, ^177^Lu, ^131^I, ^90^Sr, ^153^Sm, ^186^Re, ^32^P, ^90^Y, ^38^Cl and ^88^Rb radionuclides are presented in Table 3 (the differences ranged from 1.20% to 29.62%) and shown as a function of the generalized radius *ρ* in Figure 3. For all the radionuclides, these data were obtained from spherical, oblate and prolate ellipsoidal shapes. In Figure 3, the solid lines represent the analytical fitting of each data series with the function presented in Equation (6). In Table 4, the *s*, *ρ*_0_ and *p* fitting parameters are presented for each radionuclide. The linear regression *R*^2^ and the sum of SSE values are listed in Table 4, and these results demonstrate the goodness of fitting.

**Figure 4:**
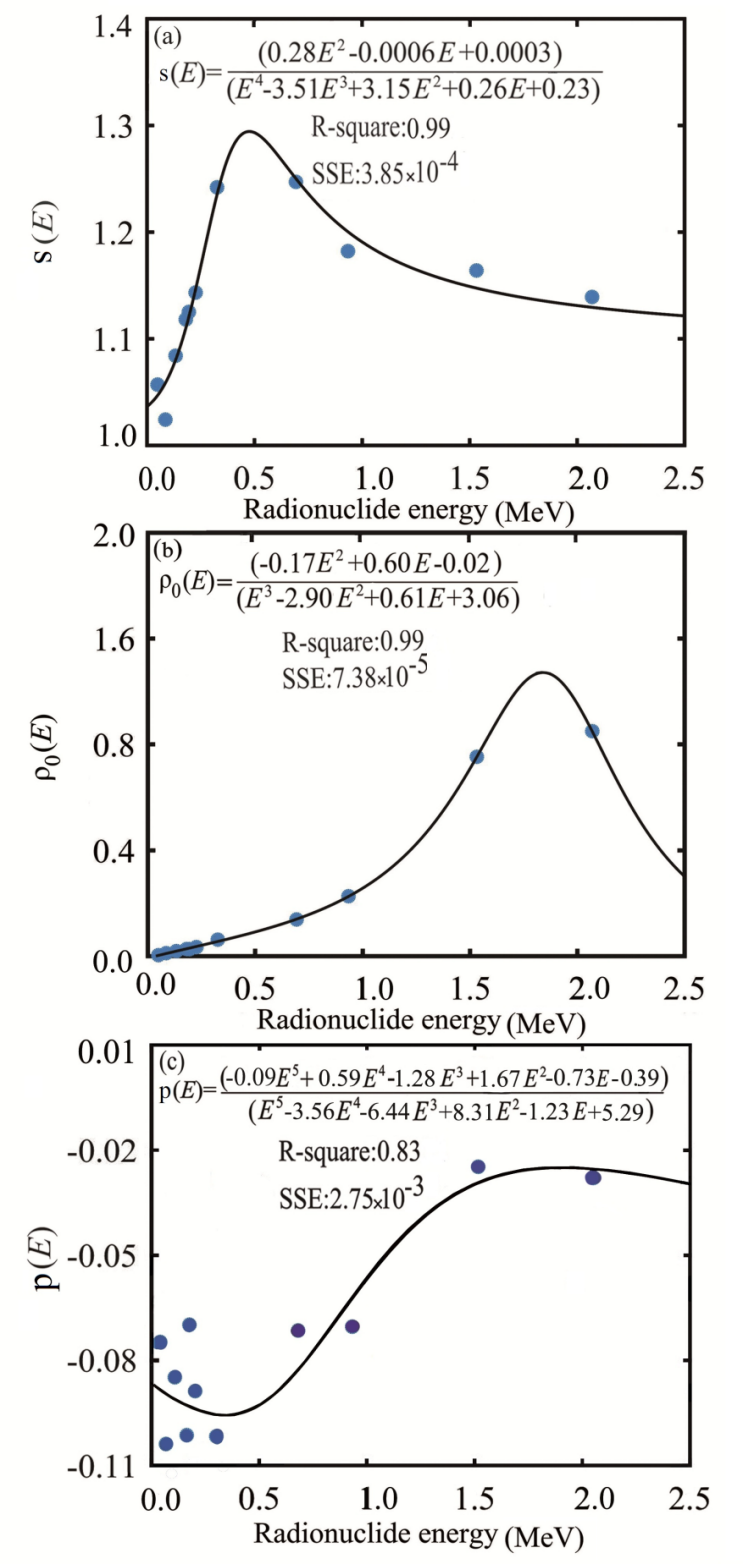
Fitting of (a)* s *(*E*), (b) *ρ*_0_(*E*) and (c) *p*(*E*) as function of radionuclide’smean energy

In Figure 4, the parameters *s, ρ*_0_ and *p* are plotted as function of the radionuclide mean beta energy. The linear regression, *R*^2^, and SSE values are also mentioned in this figure.

Table 5 presents the absorbed doses for ^14^C, ^199^Au,^177^Lu, ^131^I, ^90^Sr, ^153^Sm, ^186^Re, ^32^P, ^90^Y, ^38^Cl and ^88^Rb radionuclides calculated by Equations (2) and (6). Then these results are compared with the results obtained from the equations presented by Amato *et al*. (9). It can be seen that the maximum differences between the results of beta absorbed fraction obtained from this study and the equations presented by Amato et al. (9) are 11.11%, 24.17% and 93.36% for the 753.97, 67.03 and 6.27 cm^3^ volumes, respectively. The main differences found were subjected to a further comparison with respect to the reference values calculated by the means of the sphere model contained in the OLINDA/EXM software (19). The comparison of the results of beta absorbed dose from this study and those by Amato *et al*. (13) and the OLINDA sphere model for ^32^P, ^90^Y, ^38^Cl in 6.28 and 67.02 cm^3^ volumes are listed in Table 6. The differences in the self-dose results between the present study and this software were in the range of 0.67% to 5.11%, except for the highest energy nuclide (^38^Cl) in 6.283 cm^3^ volume.

## Discussion

In the present study, the effect of the beta spectrum on the absorbed fraction (self-absorption) was quantified for several radionuclides that are commonly used in nuclear medicine. Then, suitable analytical functions were found for the beta energy spectrum absorbed fractions in spherical and ellipsoidal volumes with uniform distribution of ^14^C, ^199^Au, ^177^Lu, ^131^I, ^90^Sr, ^153^Sm, ^186^Re, ^32^P, ^90^Y, ^38^Cl and ^88^Rb radionuclides. The validations of the simulations were based on a comparison of the simulated beta absorbed fraction for ^90^Y radionuclide in spheres with the data previously published by Akabani *et al*. (11) and Amato *et al*. (9-13) for the mean and the spectrum energy. As per the data presented in Table 2, the maximum differences in the absorbed fractions for ^90^Y radionuclide between the present study and the previous studies are 3.00% and 5.00% for the mean and the spectrum energy, respectively. Generally, these results are in good agreement with the reported data of the previous studies (9-11). The absorbed fraction depends on the size and the geometry of the source, the distribution of the radioactive material, the type of the emitted radiations, and the energies emitted by the radionuclide (11). Any change in each of these parameters will change the amount of the absorbed fraction. In the present study, the same size and geometry for the source and the distribution of radioactive material was used as they were described in previous studies (9-13). However, they were not used for the same energy spectra and this could be one of the reasons for the differences between the data presented in the present study and those in the previous studies. Phantom included separate spheres of volumes ranging from 0.004 cm^3^ to 100 cm^3^. Another reason for the differences observed in these data can be due to the differences in the Monte Carlo codes. According to the results presented by previous literature, the difference between the absorbed fractions of beta from the different versions of Monte Carlo codes ranged from 2.00% to 3.00% for low-energy and 10% for high-energy beta particles (21).This may be due to the different cross-section libraries for the various electron interactions and the different electron transport algorithms that are used in different codes. 

Table 3 indicates the importance of considering the beta spectrum as opposed to using the average beta energy in dosimetric calculations due to the differences in the calculated absorbed fractions. According to these results, the differences between the absorbed fractions from the mean and the spectrum beta energy for beta emitted by ^14^C, ^199^Au,^ 177^Lu, ^131^I, ^90^Sr, ^153^Sm, ^186^Re, ^32^P, ^90^Y, ^38^Cl and ^88^Rb ranged ranging from 1.20% to 29.62%. The maximum type A uncertainty in the Monte Carlo calculations in this step was 3.00%; this difference level between these two data series indicates the discrepancy in the calculation of the absorption fraction by applying the mean beta energy. 

**Table 1:** Element composition and density of soft tissue (14)

**Element**	**Percentage by weight (%)**
H	10.454
C	22.663
N	2.490
O	63.525
Na	0.112
Mg	0.013
Si	0.030
P	0.134
S	0.204
Cl	0.133
K	0.208
Ca	0.024
Fe	0.005
Zn	0.003
Rb	0.001
Zr	0.001
Density	1.04 g/cm^3^

**Table 2:** Absorbed fractions for ^90^Y radionuclide in this study and from previous studies

**Beta absorbed fractions (ᶲ**_β_ **)**			**Radius (cm)**	**Volume (cm** ^3^ **)**
**By mean beta energy**				
**σ(%)**	**Previous study (Reference)**	**This work**		
-1.00	0.16 (Akabani*et al*. (11))	0.15	0.10	0.004
-3.00	0.48 (Akabani*et al*. (11))	0.45	0.25	0.065
-2.00	0.86 (Akabani*et al*. (11))	0.84	1.00	4
By spectral beta energy				
-1.00	0.17 (Akabani*et al*. (11))	0.16	0.10	0.004
-3.00	0.39 (Akabani*et al*. (11))	0.36	0.25	0.065
-5.00	0.80 (Akabani*et al*. (11))	0.75	1.00	4
-5.00	0.85 (Amato *et al*. (13))	0.80	1.33	10
-2.00	0.88 (Amato *et al*. (13))	0.90	1.68	20
1.00	0.93 (Amato *et al*. (13))	0.94	2.87	100

**Table 3:** Absorbed fractions obtained by mean beta energy and spectral beta energies in spherical and elliptical volumes for^ 4^C, ^199^Au, ^177^Lu, ^131^I, ^90^Sr, ^153^Sm, ^186^Re, ^32^P, ^90^Y, ^38^Cl and ^88^Rb radionuclides

**Beta absorbed fraction ** **(ᶲ**_β_ **) **													
**Elliptical volume (a=b=2c)**				**Elliptical volume (a=b=** **c/2)**				**Spherical volume**				**Energy**	**Volume** **(cm)**
**200**	**100**	**1**	**0.01**	**200 **	**100**	**1**	**0.01**	**200**	**100**	**1**	**0.01**		
0.924 ±0.02	0.924 ±0.02	0.920 ±0.02	0.898 ±0.02	0.924 ±0.02	0.924 ±0.02	0.920 ±0.02	0.898 ±0.02	0.925 ±0.02	0.924 ±0.02	0.920 ±0.02	0.900 ±0.02	Spectrum	^14^C
0.999 ±0.01	0.999 ±0.01	0.997 ±0.01	0.985 ±0.01	0.999 ±0.01	0.999 ±0.01	0.994 ±0.01	0.985 ±0.01	1.000 ±0.01	0.999 ±0.01	0.997 ±0.01	0.986 ±0.01	Mean	
-7.51	-7.51	-7.72	-8.83	-7.51	-7.51	-7.42	-8.83	-7.50	-7.51	-7.72	-8.72	σ(%)	
0.893 ±0.03	0.893 ±0.03	0.878 ±0.03	0.814 ±0.03	0.893 ±0.03	0.893 ±0.03	0.855 ±0.03	0.815 ±0.03	0.893 ±0.03	0.892 ±0.03	0.880 ±0.03	0.821 ±0.03	Spectrum	
0.999 ±0.01	0.998 ±0.01	0.992 ±0.01	0.960 ±0.01	0.999 ±0.01	0.998 ±0.01	0.992 ±0.01	0.961 ±0.01	0.999 ±0.01	0.998 ±0.01	0.992 ±0.01	0.964 ±0.01	Mean	^199^Au
-10.61	-10.52	-11.49	-15.21	-10.61	-10.52	-13.81	-15.19	-10.61	-10.62	-11.29	-14.83	σ(%)	
0.911 ±0.03	0.910 ±0.03	0.882 ±0.03	0.761 ±0.02	0.911 ±0.03	0.910 ±0.03	0.883 ±0.03	0.763 ±0.02	0.911 ±0.03	0.910 ±0.03	0.885 ±0.03	0.774 ±0.02	Spectrum	
0.997 ±0.01	0.996 ±0.01	0.983 ±0.01	0.919 ±0.01	0.997 ±0.01	0.996 ±0.01	0.983 ±0.01	0.920 ±0.01	0.997 ±0.01	0.997 ±0.01	0.984 ±0.01	0.927 ±0.01	Mean	^177^Lu
-8.62	-8.63	-10.27	-17.19	-8.53	-8.63	-10.17	-17.06	-8.62	-8.72	-10. 06	-16.50	σ(%)	
0.893 ±0.03	0.891 ±0.03	0.853 ±0.03	0.688 ±0.02	0.893 ±0.03	0.891 ±0.03	0.854 ±0.03	0.692 ±0.02	0.893 ±0.03	0.891 ±0.03	0.857 ±0.03	0.705 ±0.02	Spectrum	
0.995 ±0.01	0.994 ±0.01	0.972 ±0.01	0.869 ±0.01	0.995 ±0.01	0.994 ±0.01	0.972 ±0.01	0.871 ±0.01	0.996 ±0.01	0.994 ±0.01	0.974 ±0.01	0.882 ±0.01	Mean	^131^I
-10.25	-10.36	-12.24	-20.82	-10.25	-10.36	-12.14	-20.55	-10.34	-10.36	-12.01	-20.06	σ(%)	
0.925 ±0.03	0.923 ±0.03	0.883 ±0.02	0.708 ±0.02	0.925 ±0.03	0.923 ±0.03	0.883 ±0.02	0.711 ±0.02	0.925 ±0.03	0.923 ±0.03	0.886 ±0.02	0.726 ±0.02	Spectrum	
0.992 ±0.01	0.990 ±0.01	0.965 ±0.01	0.850 ±0.01	0.991 ±0.01	0.990 ±0.01	0.965 ±0.01	0.852 ±0.01	0.993 ±0.01	0.991 ±0.01	0.967 ±0.01	0.865 ±0.01	Mean	^90^Sr
-6.75	-6.76	-8.49	-16.7	-6.65	-6.76	-8.49	-16.54	-6.85	-6.86	-8.37	-16.06	σ(%)	
0.903 ±0.03	0.900 ±0.03	0.849 ±0.02	0.636 ±0.02	0.904 ±0.03	0.901 ±0.03	0.850 ±0.02	0.640 ±0.02	0.904 ±0.03	0.901 ±0.03	0.855 ±0.03	0.657 ±0.02	Spectrum	
0.992 ±0.01	0.988 ±0.01	0.958 ±0.01	0.813 ±0.02	0.992 ±0.01	0.989 ±0.01	0.959 ±0.01	0.816 ±0.02	0.992 ±0.01	0.991 ±0.01	0.962 ±0.01	0.832 ±0.02	Mean	^153^Sm
-8.97	-8.91	-11.37	-21.77	-8.87	-8.89	-11.36	-21.57	-8.87	-9.08	-11.12	-21.03	σ(%)	
0.899 ±0.03	0.892 ±0.03	0.832 ±0.02	0.552 ±0.02	0.902 ±0.03	0.893 ±0.03	0.832 ±0.02	0.555 ±0.02	0.902 ±0.03	0.894 ±0.03	0.834 ±0.02	0.555 ±0.02	Spectrum	
0.988 ±0.01	0.985 ±0.01	0.930 ±0.01	0.692 ±0.02	0.988 ±0.01	0.985 ±0.01	0.932 ±0.01	0.697 ±0.02	0.989 ±0.01	0.986 ±0.01	0.937 ±0.01	0.717 ±0.02	Mean	^186^Re
-9.01	-9.44	-10.54	-20.23	-8.70	-9.34	-10.73	-20.37	-8.79	-9.33	-10.99	-22.59	σ(%)	
0.901 ±0.02	0.890 ±0.02	0.714 ±0.02	0.280 ±0.02	0.902 ±0.02	0.891 ±0.02	0.718 ±0.02	0.282 ±0.02	0.904 ±0.02	0.895 ±0.02	0.734 ±0.02	0.295 ±0.01	Spectrum	
0.967 ±0.02	0.958 ±0.02	0.810 ±0.02	0.305 ±0.02	0.967 ±0.02	0.956 ±0.02	0.813 ±0.02	0.306 ±0.02	0.969 ±0.02	0.961 ±0.02	0.827 ±0.02	0.337 ±0.01	Mean	^32^P
-6.83	-7.09	-11.85	-8.19	-6.72	-6.79	-11.68	-7.84	-6.71	-6.86	-11.24	12.46	σ(%)	
0.881 ±0.02	0.866 ±0.02	0.620 ±0.02	0.197 ±0.01	0.882 ±0.02	0.867 ±0.02	0.624 ±0.02	0.198 ±0.01	0.886 ±0.02	0.872 ±0.02	0.645 ±0.02	0.207 ±0.01	Spectrum	
0.950 ±0.02	0.931 ±0.02	0.732 ±0.02	0.204 ±0.01	0.950 ±0.02	0.935 ±0.02	0.736 ±0.02	0.204 ±0.01	0.956 ±0.02	0.946 ±0.02	0.755 ±0.02	0.215 ±0.01	Mean	^90^Y
-7.26	-6.98	-15.30	-3.43	-7.16	-7.27	-15.22	-2.94	-7.32	-7.82	-14.56	-3.72	σ(%)	
0.800 ±0.03	0.769 ±0.03	0.391 ±0.02	0.114 ±0.01	0.802 ±0.03	0.771 ±0.03	0.393 ±0.02	0.114 ±0.01	0.810 ±0.03	0.782 ±0.03	0.411 ±0.02	0.121 ±0.01	Spectrum	
0.916 ±0.02	0.894 ±0.02	0.551 ±0.02	0.112 ±0.01	0.917 ±0.02	0.896 ±0.02	0.556 ±0.02	0.112 ±0.01	0.922 ±0.01	0.903 ±0.01	0.584 ±0.02	0.119 ±0.01	Mean	^38^Cl
-12.66	-13.98	-29.04	1.785	-12.54	-13.95	-29.32	1.78	-12.14	-13.39	-29.62	1.68	σ(%)	
0.795 ±0.02	0.760 ±0.02	0.350 ±0.02	0.084 ±0.01	0.797 ±0.02	0.762 ±0.02	0.352 ±0.02	0.084 ±0.01	0.806 ±0.02	0.733 ±0.02	0.369 ±0.02	0.090 ±0.01	Spectrum	
0.884 ±0.02	0.855 ±0.02	0.425 ±0.02	0.083 ±0.02	0.886 ±0.02	0.857 ±0.02	0.428 ±0.02	0.083 ±0.01	0.894 ±0.02	0.866 ±0.02	0.457 ±0.02	0.088 ±0.01	Mean	^88^Rb
-10.06	-11.11	-17.65	1.20	-10.04	-11.08	-17.75	1.20	-9.84	-15.35	-19.25	2.27	σ(%)	

**Table 4:** *s*(*E*),* ρ*_0_(*E*) and *p*(*E*) fitting parameters,* R*^2 ^and SSE values obtained from equations (6) and equations presented by Amato *et al*. (9)

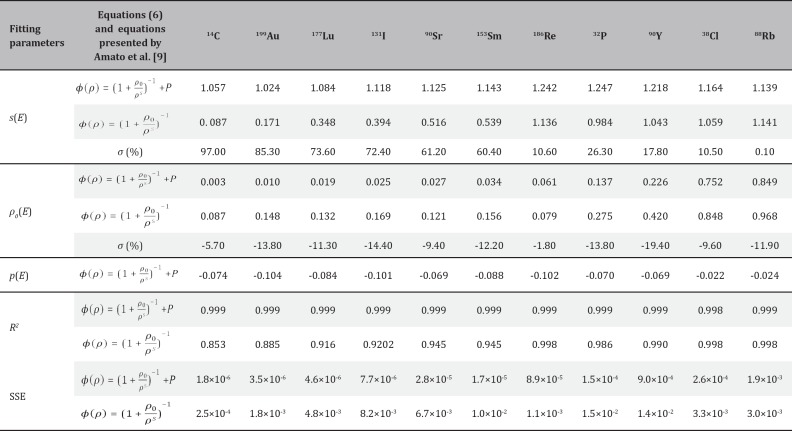

**Table 5:** Comparison of the results of beta absorbed dose from this study and those by equations presented by Amato et al. (9)

**Absorbed dose (µGy/MBqs)**									
**Volume=6.28 (cm** ^3^ **)**			**Volume=67.03 (cm** ^3^ **)**			**Volume=753.97 (cm** ^3^ **)**			**Radionuclide**
**Diff. (%)**	**Equations presented by Amato et al. **	**This study**	**Diff. (%)**	**Equations presented by Ama**	**This study**	**Diff. (%)**	**Equations presented by Amato et al. **	**This study**	
-6.61	2.706	2.527	-8.49	0. 259	0. 237	-8.69	0.023	0.021	^14^C
8.57	2.123	1.941	-9.00	0. 200	0. 182	-11.11	0.018	0.016	^199^Au
-7.87	3.212	2.959	-9.18	0. 305	0. 277	-7.41	0.027	0.025	^177^Lu
-7.00	4.341	4.037	-8.91	0. 415	0. 378	-8.11	0. 037	0.034	^131^I
-6.99	4.673	4.346	-8.94	0. 447	0. 407	-10.00	0.040	0.036	^90^Sr
-5.92	5.368	5.050	-8.68	0. 518	0. 473	-8.69	0.046	0.042	^153^Sm
-2.31	7.450	7.278	-7.58	0. 738	0. 682	-8.95	0.067	0.061	^186^Re
9.03	14.102	15.376	-4.37	1.507	1.441	-8.57	0. 140	0. 128	^32^P
21.44	17.024	20.675	-0. 51	1.948	1.938	-7.02	0. 185	0. 172	^90^Y
73.08	19.584	33.897	18.15	2.689	3.177	-1.05	0. 285	0. 282	^38^Cl
93.36	23.641	45.714	24.17	3.451	4.285	-1.06	0. 377	0. 381	^88^Rb

**Table 6:** Comparison of the results of beta absorbed dose from this study and those by equations presented by Amato et al. (9) and from OLINDA/EXM software (sphere model) for ^32^P, ^90^Y, ^38^Cl in 6.283 cm^3^and 67.021 cm^3^ volumes

**Absorbed dose (µGy/MBqs)**						
**Volume=67.02cm** ^3^						**Radionuclide**
**Diff. (%)**	**OLINDA**	**equations presented by Amatoet al.[9]**	**Diff. (%)**	**OLINDA**	**This study**	
-19.21	3.33	2.69	-4.50	3.33	3.18	^38^Cl
Volume=6. 28 cm^3^						
-12.96	16.20	14.10	-5.11	16.20	15.38	^32^P
-18.21	20.81	17.02	-0.67	20.81	20.67	^90^Y
-31.05	28.40	19.58	16.90	28.40	33.20	^38^Cl

The absorbed fraction can be defined as the fraction of the energy emitted in the source volume that was absorbed in the target volume (10). The beta particle interacts with the atoms of the material through inelastic collisions with atomic electrons (excitation and ionization), elastic scattering by nuclei, bremsstrahlung and emission of Cherenkov radiation depending on their energy. Due to the continuous energy distribution of the beta particles, the absorption of the beta particles in the material is also continuous (22). When the monoenergetic electrons interact with the matter, bremsstrahlung and excitation interactions occur with rates that depend on the electron energy. On the other hand, when poly-energetic beta particles interact with the atoms, the probabilities of various interactions vary with the particle energy. The use of either mean or mono-energy spectrum for the beta particles can have an effect on the absorbed fraction results and therefore on the absorbed dose (10). This is because the cross-sections of different electron collisions depend on the radiant energy of the radionuclide. According to the results of the present study, considerable differences were observed between the absorbed fractions obtained by the mean energy and the discrete spectrum energies.

The information presented in Figure 3 shows that for each radionuclide analyzed and for each geometrical configuration (sphere, oblate ellipsoid, and prolate ellipsoid), the absorbed fraction is a function of the generalized radius. The perceivable dependence of ϕ on *ρ* is due to the fraction of the beta particles escaping from the target volume, and this fraction depends on the volume-surface ratio (9-13). 

Amato *et al*. (9-13) suggested an analytical function for the relationship between the absorbed fraction and the generalized radius (*ρ*), providing the parameters for some extended beta spectra that are commonly employed in nuclear medicine (^199^Au, ^177^Lu, ^131^I, ^153^Sm, ^186^Re and ^90^Y) as reference (13) and then extending their analytical formalism to a generic beta spectrum through integration (9). Regarding the nuclides considered in the present study, i.e., ^14^C, ^199^Au, ^177^Lu, ^131^I, ^90^Sr, ^153^Sm, ^186^Re, ^32^P, ^90^Y, ^38^Cl and ^88^Rb, the differences between the *ρ*_0 _and *s *fitting parameters obtained from Equation (6) and the equations presented by Amato *et al. *(9) are presented in Table 4. The linear regression, *R*^2^, and SSE values are also presented in this table. According to the data provided for *R*^2^ and SSE, it can be concluded that the linear fitting function in Equation (6) is a more accurate choice for calculating the absorbed fraction of the beta spectrum.

Figure 4 shows no constant relationship between the exponent *s*and *p*, and the beta energy of the radionuclides. On the contrary, *ρ*_0_ is proportional to the beta energy of the radionuclide. In order to calculate the absorbed fraction by spectral energies there is a need to calculate s, ρ_0_ and *p* from the equations shown in Figure 4.

The absorbed fraction can be obtained for each generalized radius by substituting *s, ρ*_0_ and *p *in Equation (6). And finally, the absorbed dose can be calculated by Equation (2). Table 5 presents the results of the comparison between radiation absorbed doses calculated by means of our analytical approach, and the tabular values obtained from the analytical approach presented by Amato *et al*. (9) for ^14^C, ^199^Au, ^177^Lu, ^131^I, ^90^Sr, ^153^Sm, ^186^Re, ^32^P, ^90^Y, ^38^Cl and ^88^Rb radionuclides. These data are related to volumes of 753.984 cm^3^, 67.021 cm^3^, and 6.283 cm^3^. According to this table, the relative difference of the absorbed doses between the present study and the previous study ranged from 0.51% to 93.36%.These data indicate that the beta particle’s spectrum has a considerable effect on the radiation absorbed dose in various volumes, especially in small volumes. With an increase in the beta energy, this relative difference is increased. As it can be seen from the data in Table 5, the maximum relative difference for the absorbed dose between this study and the previous work is 93.36% for ^88^R bradionuclide in a volume of 6.283 cm^3^. The cause for these differences may be due to the differences in the analytical functions employed, the shape of the actual beta spectra used, and the Monte Carlo codes employed.

Taking OLINDA/EXM software as a reference, the differences in the self-dose results between the present study and this software were in the range of 0.67% to 5.11%, except for the highest energy nuclide (^38^Cl) in a volume of 6.283 cm^3^as reported in Table 6. These discrepancies could be once again at tribute to the slightly different emission spectra simulated, and to the simulation codes. Generally, these results were in good agreement with the reference data. 

The present study evaluated the influence of the discrete spectral definition of beta energy on the absorbed fraction. In all the steps of the calculations, the spectrum was considered for beta particles, except for the fitting parameters, for which the mean energy was considered for the beta particles. For future studies, it is suggested that the spectrum beta should be used for all the steps, including the fitting process. In this study a discrete spectrum was applied for the beta particles of various radionuclides. To increase the accuracy in calculating the absorbed dose, it is recommended that a continuous spectrum should be used for the beta particles and this may be a subject for further research in this field. 

## Conclusion

This work developed a new analytical model, which enables more accurate calculations of the absorbed fractions for beta emitting radionuclides. The results obtained from the validations of the simulations were in good agreement with the data reported in previous studies. Moreover, the agreement between this study and the OLINDA/EXM data once again demonstrated the validity of the results of the present analytical model. 

The International Commission on Radiation Units and Measurements (ICRU) in report No. 24 recommended that the uncertainty in radiation dose delivery in radiotherapy should be in the range of ±5% (23). Based on the results of this study, the differences between the calculated absorbed fraction by average and spectrum beta energies ranged from 0.120% to 29.62%. Therefore, the impact of the spectral energy on the absorbed fraction on the MIRD formulas should be corrected in order to minimize the total uncertainty in dose delivery in internal radiotherapy. 

The use of the analytical formulas presented for the absorbed fraction as a function of the generalized radius, and the parametric functions for *ρ*_0_ (*E*), *s* (*E*) and *p* (*E*), enables a detailed calculation of the absorbed dose for the whole energy range of practical interest for internal dosimetry in nuclear medicine therapeutic applications and radiological protection estimations of doses from internal pollutions. The parameters in the presented formulas depend on the radiation energy spectrum and the radionuclide energy. 
